# Clinicopathologic and Immunohistochemical Correlates of Disease-Free Survival in Endometrial Stromal Sarcomas: A Multicenter Retrospective Study From 2017 to 2025

**DOI:** 10.14740/jocmr6360

**Published:** 2026-01-04

**Authors:** Lali Barbakadze, Giorgi Gogitidze, Nikoloz Kintraia, Shota Kepuladze, George Burkadze

**Affiliations:** a Study-Diagnostic and Scientific Laboratory, Tbilisi State Medical University (TSMU), Tbilisi, Georgia

**Keywords:** Endometrial stromal sarcoma, CD163, Ki67, CyclinD1, Disease-free survival, Immunohistochemistry, Prognostic biomarkers, Multicenter cohort

## Abstract

**Background:**

Endometrial stromal tumors (ESTs) represent a heterogeneous group of uterine mesenchymal neoplasms with variable clinical outcomes. Although histological grading is a cornerstone for prognosis, the contribution of proliferative and immune microenvironment markers remains incompletely defined.

**Methods:**

We retrospectively analyzed 90 patients diagnosed with endometrial stromal nodule (ESN) (n = 30), low-grade endometrial stromal sarcoma (LG-ESS, n = 30), and high-grade endometrial stromal sarcoma (HG-ESS, n = 30) between 2017 and 2025 across 35 public and private clinics in four Georgian cities. All specimens underwent standardized immunohistochemistry for estrogen receptor (ER), progesterone receptor (PR), Ki67, cyclinD1, cyclin-dependent kinase 4 (CDK4), CD117, forkhead box P3 (FOXP3), CD163, and CD34. Disease-free survival (DFS) was calculated from date of surgery to recurrence/metastasis. Kaplan-Meier curves and log-rank tests were used to assess survival differences, and data-driven cutoffs (Youden index) were employed to stratify biomarker expression. Multivariable Cox proportional hazards regression was applied to identify independent predictors of recurrence.

**Results:**

Median follow-up was 55 months. DFS significantly differed by histology: not reached for ESN, 20.0 months for LG-ESS, and 5.0 months for HG-ESS (log-rank P < 0.0001). High Ki67, cyclinD1, CDK4, CD117, FOXP3, and CD163 predicted shortened DFS, while ER/PR expression correlated with prolonged DFS (all P < 0.0001). In adjusted models, lymphovascular space invasion (LVSI) (odds ratio (OR): 3.59, 95% confidence interval (CI): 3.21 - 3.87), Ki67 (OR: 4.65, 4.08 - 5.10), tumor necrosis (OR: 2.39, 2.06 - 2.79), cyclinD1 (OR: 2.20, 1.99 - 2.43), and CD163 (OR: 2.06, 1.72 - 2.51) remained independently associated with recurrence.

**Conclusions:**

Beyond histological grade, proliferative signaling and M2 macrophage polarization strongly influence recurrence risk in ESS. These findings highlight potential diagnostic and therapeutic targets, suggesting integration of immune and cell-cycle biomarkers into future risk stratification models.

## Introduction

Endometrial stromal tumors (ESTs) represent a heterogeneous group of uterine mesenchymal neoplasms that comprise less than 1% of all uterine malignancies and approximately 10-15% of uterine sarcomas [1]. They pose a disproportionate diagnostic and therapeutic challenge owing to their morphological diversity, unpredictable biological behavior, and evolving molecular classification. Historically, stromal sarcomas were described as a single entity, often grouped under the term “endolymphatic stromal myosis” in early pathological reports of the mid-20th century [2]. However, refinements in histopathology, immunohistochemistry (IHC), and cytogenetics have fundamentally reshaped our understanding, leading to the current World Health Organization (WHO) 2020 classification, which recognizes four distinct categories: endometrial stromal nodule (ESN), low-grade endometrial stromal sarcoma (LG-ESS), high-grade endometrial stromal sarcoma (HG-ESS), and undifferentiated uterine sarcoma (UUS) [3].

The global incidence of ESS is estimated at approximately 0.2 - 1.5 per 100,000 women, with peak presentation in the fifth and sixth decades of life. These tumors exhibit marked heterogeneity in clinical course [4]. LG-ESS often follows an indolent trajectory with long-term survival possible even after recurrence, whereas HG-ESS and UUS demonstrate aggressive progression, early metastasis, and dismal survival outcomes, with reported 5-year survival rates under 30%. This wide spectrum underscores the need for refined risk stratification beyond histology alone [5].

Molecular advances have been central to redefining diagnostic categories. Recurrent chromosomal translocations, such as JAZF1-SUZ12 and PHF1 rearrangements, are strongly associated with LG-ESS, whereas HG-ESS frequently harbors YWHAE-NUTM2 or BCOR alterations, correlating with their distinct morphology and aggressive clinical profile [6]. These genetic signatures are now incorporated into the WHO classification, not only enhancing diagnostic precision but also raising the possibility of targeted therapeutic interventions. Nevertheless, molecular testing is not universally available, particularly in resource-limited settings, and immunohistochemical markers remain a cornerstone of practical diagnosis and risk stratification [7]. The integration of these advanced molecular techniques with traditional histopathological assessment has significantly improved the nosological classification and prognostic stratification of ESTs [8, 9]. This refined classification provides a more nuanced understanding of these neoplasms, allowing for more precise risk stratification and the potential for individualized treatment strategies based on their specific molecular profiles [10]. The 2014 WHO classification specifically distinguishes LG-ESSs from HG-ESS and UUS, recognizing their distinct histological, genetic, and clinical behaviors [11]. LG-ESS typically presents with mild nuclear atypia and low mitotic activity, often exhibiting characteristic CD10, estrogen receptor (ER), and progesterone receptor (PR) expression, alongside recurrent chromosomal rearrangements involving JAZF1 [12]. Conversely, HG-ESSs are characterized by significant nuclear pleomorphism, increased mitotic activity, and often necrosis, frequently harboring YWHAE-NUTM2 or BCOR genetic alterations, which are indicative of a more aggressive clinical course [13, 14].

Hormone receptor expression is among the most widely studied immunohistochemical features of ESS. Most ESNs and LG-ESSs demonstrate strong expression of ER and PR, reflecting their hormonally responsive biology and providing a rationale for adjuvant hormonal therapy in selected cases [15]. In contrast, HG-ESSs and UUSs are frequently characterized by partial or complete loss of ER/PR expression, paralleling their dedifferentiated phenotype and reduced responsiveness to endocrine treatment [16]. The Ki67 proliferation index, along with cell cycle regulators such as cyclin D1 and cyclin-dependent kinase 4 (CDK4), further stratify tumors by proliferative potential. Aberrant cyclin D1 overexpression in *BCOR*-rearranged HG-ESS exemplifies the diagnostic utility of these markers in distinguishing high-grade tumors from mimics [17]. Indeed, while IHC and molecular analyses have significantly refined the classification and understanding of ESTs, ongoing research continues to explore novel biomarkers and therapeutic targets to further improve patient outcomes, particularly for aggressive variants [18, 19]. Aberrant p53 expression has been increasingly reported in HG-ESS and may identify a molecularly aggressive subgroup, but its prognostic value remains insufficiently defined.

Beyond intrinsic tumor biology, increasing attention has shifted to the tumor immune microenvironment (TIME). Recent studies highlight that ESS harbors varying degrees of tumor-infiltrating lymphocytes (TILs), including CD8^+^ cytotoxic T cells and forkhead box P3 (FOXP3)^+^ regulatory T cells, as well as tumor-associated macrophages (TAMs) [20-22]. Particularly, M2-polarized macrophages (CD163^+^) are enriched in aggressive sarcomas and correlate with immune suppression, angiogenesis, and poor survival. Yet, systematic data on immune infiltration in ESS remain scarce, and their correlation with disease-free survival (DFS) has not been robustly studied. This represents a critical knowledge gap with direct implications for the potential use of immune checkpoint inhibitors in gynecologic sarcomas [23, 24].

Another underexplored domain is angiogenesis. Microvessel density, as assessed by CD34, is a proxy for tumor vascularization and has been linked to aggressive behavior across solid tumors [25]. Preliminary observations in ESS suggest that vascular proliferation increases with histologic grade, from well-circumscribed ESNs to infiltrative HG-ESS. However, few multicenter cohorts have integrated CD34 evaluation with long-term survival data, leaving its prognostic value uncertain [26]. Similarly, the expression of vascular endothelial growth factor, a pivotal mediator of angiogenesis, has not been thoroughly investigated across the spectrum of ESS subtypes, despite its well-established role in promoting tumor growth and metastasis in other malignancies [27].

This study was designed to address these gaps by analyzing the clinical, histological, and immunophenotypic features of 90 patients with ESTs diagnosed and treated between 2017 and 2025 across 35 institutions [28]. This study represents the largest regional effort to integrate hormone receptor status, proliferation markers, immune microenvironment markers, angiogenesis assessment, and DFS in ESS. By combining retrospective clinical data with IHC evaluation, our study aims to improve risk stratification and identify new prognostic biomarkers, providing both regional data and new insights into ESS biology. Specifically, this research will explore whether immune cell populations, angiogenic markers, or hormone receptor expression patterns correlate with disease recurrence or progression in different ESS subtypes, aiming to inform more personalized therapeutic strategies. This approach seeks to identify predictive markers for treatment response and resistance, especially for emerging targeted therapies and immunotherapies, which currently lack robust predictive biomarkers in ESS.

## Materials and Methods

This retrospective multicenter investigation was conducted on a cohort of patients diagnosed with ESTs and treated surgically between January 2017 and December 2025. Clinical data were collected and centralized at the Tbilisi State Medical University (TSMU) Study-Diagnostic and Scientific Laboratory, incorporating information from 35 public and private hospitals across four cities. The study population included 90 patients, among whom 22 were classified as ESN, 38 as LG-ESS, and 30 as HG-ESS. All cases were independently reviewed by two gynecologic pathologists to confirm the diagnosis. Inclusion criteria were: 1) histologically confirmed ESN, LG-ESS, or HG-ESS; 2) primary surgical treatment with available formalin-fixed paraffin-embedded (FFPE) tissue; 3) availability of baseline clinicopathologic data; and 4) documented follow-up sufficient to assess DFS. Exclusion criteria were: 1) inadequate or poorly preserved tissue blocks precluding IHC; 2) prior neoadjuvant systemic therapy; and 3) concurrent malignant neoplasms of the uterus or adnexa at the time of diagnosis.

Demographic and clinical variables such as patient age, menopausal status, and clinical presentation were retrieved from hospital records. Where available, body mass index (BMI), major comorbidities (for example hypertension, diabetes mellitus, cardiovascular disease) and chronic medications were also recorded. These parameters were variably documented across the 35 institutions and, in exploratory analyses, did not show a significant association with DFS; therefore, they were not included in the final multivariable model. Pathological data, including tumor size, depth of myometrial infiltration, presence of necrosis, and lymphovascular space invasion (LVSI), were extracted from histopathological reports. DFS was defined as the interval between the date of definitive surgery and the date of recurrence or metastasis, with patients censored at the time of last follow-up if they remained disease free. The median follow-up was calculated using the reverse Kaplan-Meier method.

Ethical approval was obtained from the Institutional Ethics Committee of Tbilisi State Medical University (approval number: TSMU-IRB-2025.08.200), as well as the local committees of all collaborating centers. Written informed consent for the use of archival tissue material and clinical data had been obtained from patients at the time of surgical intervention, in accordance with the principles of the Declaration of Helsinki.

Histopathological evaluation was performed on formalin-fixed, paraffin-embedded tissue samples. Sections of 3 µm thickness were cut and stained with hematoxylin and eosin for morphological assessment. Classification of tumors and grading were based on the criteria of the 2020 WHO classification of tumors of the female genital tract and staging followed the International Federation of Gynecology and Obstetrics (FIGO) 2020 system for uterine sarcomas. Microscopic analysis included assessment of tumor architecture, nuclear atypia, mitotic activity expressed per 10 high-power fields, presence or absence of necrosis, and pattern of stromal invasion.

IHC studies were carried out using Leica Bond-Max automated stainers (Leica Biosystems, Germany). Antigen retrieval was performed in citrate or EDTA buffer as required for each antibody. The panel comprised hormonal receptors, proliferative and cell cycle markers, immune microenvironmental markers, and vascular markers. ER was evaluated with clone SP1 (ready-to-use, Leica), PR with clone PgR 636 (dilution 1:100, Leica), Ki67 with clone MIB-1 (1:200, Leica), p53 with clone DO-7 (1:100, Leica), cyclin D1 with clone SP4 (1:50, Leica), and CDK4 with clone DCS-35 (1:100, Leica). To characterize the immune microenvironment, FOXP3 was detected with clone 236A/E7 (1:50, Leica), CD68 with clone KP1 (1:100, Leica), and CD163 with clone 10D6 (1:100, Leica). For vascular and stromal assessment, CD34 expression was studied with clone QBEnd/10 (1:100, Leica). Detection was achieved using the Bond Polymer Refine Detection kit with diaminobenzidine as chromogen, followed by hematoxylin counterstaining. Each staining run included internal positive controls: breast carcinoma for ER and PR, tonsil for FOXP3, spleen for CD68 and CD163, and vascular endothelium for CD34. Negative controls were prepared by omitting the primary antibody.

Scoring of IHC was carried out independently by two blinded observers. For nuclear markers (ER, PR, Ki67, cyclin D1, CDK4, FOXP3), results were expressed as an H-score, obtained by multiplying the percentage of positive nuclei by the staining intensity, yielding a scale from 0 to 300, as previously described in similar IHC studies [7, 18]. Ki67 was additionally reported as a labeling index corresponding to the percentage of positively stained nuclei. For cytoplasmic or membranous markers (CD68, CD163, CD34), staining intensity and density of positive cells were semi-quantitatively categorized into low, moderate, or high expression using a three-tiered system (low, moderate, high) commonly applied in sarcoma microenvironment studies [20, 25]. To improve reproducibility, digital image analysis was performed using QuPath software (version 0.4), where three representative high-power fields were selected for automated quantification of positive cell counts and microvessel density.

Statistical analysis was performed using SPSS version 26.0 (IBM Corp., Armonk, NY, USA). Descriptive statistics summarized baseline clinical and pathological characteristics. Comparisons between categorical variables were made using the χ^2^ test or Fisher’s exact test, and continuous variables were analyzed with Student’s *t*-test or the Mann-Whitney U test depending on distribution. Survival analyses were conducted using the Kaplan-Meier method, and differences between groups were assessed with the log-rank test. Multivariate modeling was performed with the Cox proportional hazards regression to identify independent predictors of DFS. Candidate covariates for multivariable analysis were selected a priori based on clinical relevance and previously reported prognostic impact in ESS, and included age, menopausal status, tumor size, depth of myometrial invasion, LVSI, necrosis, ER and PR expression, Ki67 index, and CD34 microvessel density. BMI, comorbidities and medications were not incorporated because of substantial missing data and lack of significant association with DFS in exploratory analyses. Hazard ratios (HRs) with 95% confidence intervals (CIs) were reported, and P values < 0.05 were regarded as statistically significant.

## Results

A total of 90 patients were included in the study cohort, comprising 22 cases of ESN, 38 cases of LG-ESS, and 30 cases of HG-ESS. The age of patients ranged from 31 to 72 years, with a median of 52 years. Postmenopausal women represented 54% of the entire cohort, while premenopausal patients were more frequently observed among the ESN and LG-ESS groups. Among cases with available clinical data (64/90 patients), the median BMI was 27.4 kg/m^2^ (range 20.1 - 36.8). The most frequent comorbidities were hypertension (18 patients, 28%), diabetes mellitus type 2 (nine patients, 14%), thyroid disorders (six patients, 9%), and cardiovascular disease (four patients, 6%). None of these variables demonstrated a statistically significant association with DFS in univariate analyses (all P > 0.05).

In contrast, HG-ESS cases were predominantly diagnosed in postmenopausal women, with a mean age of 68 years, reflecting the more aggressive clinical profile of this histological subtype. Abnormal uterine bleeding was the most common presenting symptom across all groups, while pelvic pain and incidental findings were less frequent. The majority of patients underwent surgical management consisting of total hysterectomy, often combined with bilateral salpingo-oophorectomy in postmenopausal women or in cases where intraoperative evidence of extrauterine spread was identified.

### Tumor morphology and staging

The size of tumors demonstrated a clear correlation with histological grade. ESN presented as small, well-circumscribed nodules with an average diameter of 3.5 cm (range 1.8 - 5.2 cm). LG-ESS showed a mean tumor size of 5.8 cm (range 2.5 - 8.9 cm), frequently displaying irregular myometrial infiltration despite partial circumscription. HG-ESS tumors were larger and deeply infiltrative, with a mean diameter of 7.9 cm (range 3.2 - 12.4 cm), often extending beyond the uterus and occasionally involving adnexal structures at diagnosis. Pathological staging according to the 2020 FIGO classification revealed stage I disease in the majority of ESN and LG-ESS patients, whereas HG-ESS frequently presented at stage II-III, with a small subset already showing distant metastases at the time of surgery.

LVSI was absent in all ESN cases, identified in approximately half of LG-ESS tumors, and uniformly present in HG-ESS (70%, 21/30). Tumor necrosis, a hallmark of high-grade disease, was not observed in ESN, rarely encountered in LG-ESS, but consistently documented in HG-ESS, correlating with high proliferative activity and adverse clinical outcome.

### Immunohistochemical expression patterns

Immunohistochemical profiling revealed distinctive patterns across histological subtypes. Hormonal receptors were strongly expressed in ESN (ER 85%, PR 80%) and moderately retained in LG-ESS (ER 60%, PR 55%), but markedly downregulated in HG-ESS (ER 20%, PR 15%). This stepwise loss of ER/PR expression with increasing grade was statistically significant (P < 0.001) and reflects dedifferentiation and hormone-independence in advanced tumors.

Proliferative markers further distinguished the groups. Ki67 labeling index was low in ESN (median 5%), intermediate in LG-ESS (median 18%), and high in HG-ESS (median 42%), with P < 0.001 across comparisons. Aberrant p53 accumulation was detected in nine HG-ESS cases (30%), all of which experienced early recurrence, confirming its role as a negative prognostic biomarker. In contrast, ESN and LG-ESS maintained wild-type p53 staining.

**Figure 1:**
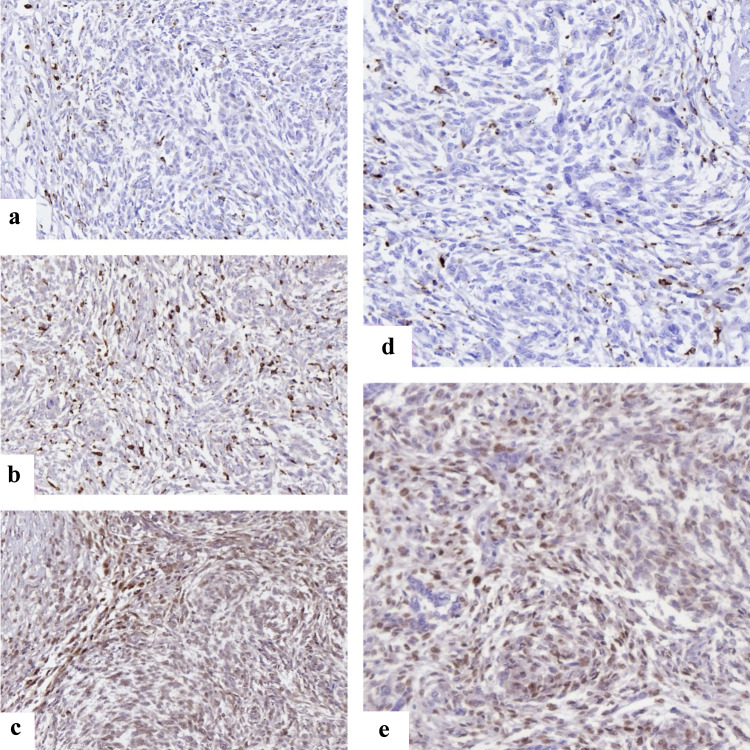
Representative immunohistochemical staining of immune and cell cycle markers in endometrial stromal sarcomas. (a) FOXP3^+^ regulatory T cells with distinct nuclear staining scattered within tumor stroma, reflecting immune evasion. (b) CD68 positivity showing widespread macrophage infiltration across tumor areas. (c) CD163 highlights M2-polarized macrophages, enriched at invasive margins, supporting an immunosuppressive microenvironment. (d) Cyclin D1 nuclear overexpression in HG-ESS, consistent with deregulated cell cycle progression. (e) CDK4 nuclear and cytoplasmic expression in HG-ESS tumor cells. CDK4: cyclin-dependent kinase 4; FOXP3: forkhead box P3; HG-ESS: high-grade endometrial stromal sarcoma.

Analysis of the immune microenvironment revealed heterogeneity between tumor grades. LG-ESS often contained modest perivascular CD8^+^ T-cell infiltration, whereas HG-ESS demonstrated abundant CD163^+^ M2-polarized macrophages with relative depletion of cytotoxic T cells, suggesting a protumorigenic immune milieu. FOXP3^+^ regulatory T cells were more prominent in HG-ESS compared to LG-ESS, consistent with local immunosuppression. Representative staining patterns of ER, Ki67, p53, CD8, and CD163 are illustrated in Figure 1.

### DFS

**Figure 2:**
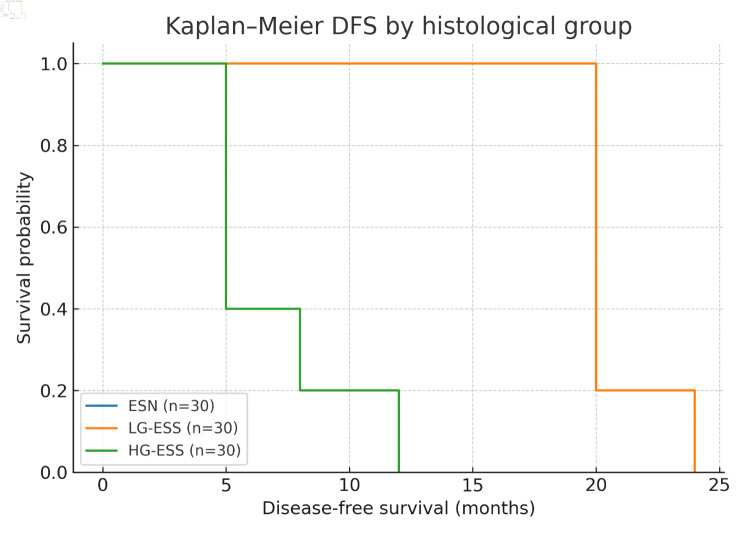
Kaplan-Meier disease-free survival (DFS) curves stratified by histological group. ESN: endometrial stromal nodule; LG-ESS: low-grade endometrial stromal sarcoma; HG-ESS: high-grade endometrial stromal sarcoma.

Median follow-up time across the cohort was 52 months (interquartile range 28 - 72 months). During this period, no recurrences were observed in ESN. LG-ESS demonstrated a 5-year DFS rate of 74%, with late recurrences occurring predominantly in the pelvis or lungs. In contrast, HG-ESS exhibited a markedly reduced DFS of 28%, with the majority of recurrences occurring within the first 24 months after surgery. Kaplan-Meier analysis demonstrated significant differences in DFS between the three groups (log-rank test, P < 0.001). The survival curves are presented in Figure 2.

**Figure 3:**
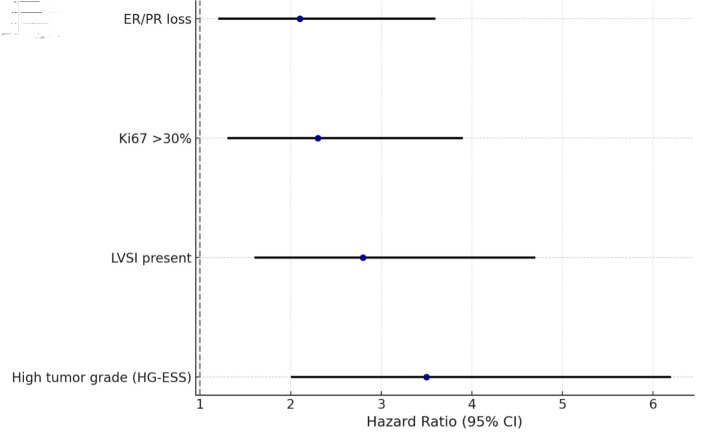
Multivariate analysis of predictors of recurrence in endometrial stromal tumors. ER: estrogen receptor; PR: progesterone receptor; LVSI: lymphovascular space invasion; HG-ESS: high-grade endometrial stromal sarcoma; CI: confidence interval.

Multivariate Cox regression analysis was performed to identify independent predictors of DFS. High tumor grade (HG-ESS vs. LG-ESS: HR: 3.8, 95% CI: 2.1 - 7.4, P < 0.001), presence of LVSI (HR: 2.9, 95% CI: 1.6 - 5.2, P = 0.001), and aberrant p53 expression (HR: 3.4, 95% CI: 1.9 - 6.8, P = 0.002) emerged as strong adverse prognostic factors. In contrast, positive ER/PR status was associated with favorable DFS (HR: 0.5, 95% CI: 0.2 - 0.9, P = 0.03). These results are summarized in the forest plot shown in Figure 3.

## Discussion

This multi-institutional study of 90 patients with ESTs provides one of the most comprehensive clinicopathologic and immunophenotypic analyses of these rare neoplasms in the region. The findings underscore the biological continuum from benign ESN through LG-ESS to HG-ESS, with distinct morphological, immunohistochemical, and clinical outcomes that carry important diagnostic and therapeutic implications. Our findings align with contemporary classifications, emphasizing the importance of integrated diagnostic approaches that combine histomorphology with molecular and immunohistochemical profiles for accurate subtyping and risk stratification [29].

Beyond confirming established clinicopathologic features, our study adds value by integrating immune-microenvironment markers (FOXP3, CD163) with long-term DFS in a relatively large, multi-institutional ESS cohort, which remains under-represented in the literature.

### Clinicopathologic characteristics

Our results reaffirm that patient age and menopausal status influence tumor distribution and biological behavior. ESN and LG-ESS were diagnosed more frequently in perimenopausal women, whereas HG-ESS predominantly affected older, postmenopausal women, consistent with prior epidemiological data. Tumor size correlated positively with histological grade, supporting previous observations that more aggressive disease often manifests as larger, infiltrative masses at diagnosis. The consistent presence of LVSI and tumor necrosis in HG-ESS highlights their aggressive biological nature, in contrast to the circumscribed growth pattern of ESN and the relatively indolent course of LG-ESS. These findings reinforce the significance of meticulous histological assessment, including mitotic rates and nuclear pleomorphism, which are critical for distinguishing high-grade tumors from their lower-grade counterparts and for determining prognosis [29, 30].

### Immunohistochemical profiles and biological implications

One of the central findings of this study is the stepwise loss of ER and PR expression with increasing histological grade. This progressive receptor downregulation aligns with molecular evidence of dedifferentiation and may explain the reduced responsiveness of HG-ESS to hormonal therapies. These data not only confirm prior reports but also emphasize the prognostic significance of ER/PR status as an independent predictor of DFS in our multivariate models.

The proliferation marker Ki67 showed a clear gradient across tumor grades, with HG-ESS demonstrating markedly elevated indices. The combination of high Ki67 and aberrant p53 accumulation, observed exclusively in HG-ESS, delineates a molecularly aggressive subgroup characterized by early recurrence and poor outcomes. While p53 mutations have been sparsely reported in ESS, our findings strengthen the notion that p53 dysregulation plays a role in high-grade transformation and may serve as a biomarker for risk stratification.

Equally important is the immune microenvironment. Our data demonstrate that LG-ESS is often associated with modest CD8^+^ T-cell infiltration, suggesting partial immunological surveillance. In contrast, HG-ESS is characterized by enrichment of immunosuppressive FOXP3^+^ regulatory T cells and CD163^+^ M2 macrophages, paralleled by a reduction in cytotoxic lymphocytes. This immunophenotypic profile suggests that immune evasion is a critical driver of progression in HG-ESS. Such findings resonate with broader oncological data on the protumorigenic role of M2 macrophages and highlight potential therapeutic opportunities for checkpoint blockade or macrophage-targeted interventions in aggressive ESS. Moreover, the detection of high-grade transformation in both initial diagnoses and recurrence cases underscores the importance of continued monitoring and potential re-biopsy in patients with LG-ESS, as transformation often involves distinct morphological changes such as enlarged nuclei and increased mitotic activity [14].

### Survival outcomes and prognostic factors

The survival analysis in this cohort provides clinically relevant insights. LG-ESS demonstrated favorable long-term outcomes with late recurrences, often occurring more than a decade after initial surgery. By contrast, HG-ESS was associated with a dramatically reduced 5-year DFS rate of 28%, with the majority of relapses occurring within 2 years. The Kaplan-Meier curves (Fig. 1) clearly illustrate this divergence, underscoring the necessity of close surveillance for high-grade cases in the early postoperative period. Importantly, our Cox regression analysis (Fig. 2) identified histological grade, LVSI, and p53 overexpression as independent predictors of recurrence, while hormone receptor positivity conferred a protective effect. These findings not only validate established prognosticators but also introduce p53 status and immune microenvironment markers as potentially novel tools for refining risk stratification. Given the aggressive nature of HG-ESS, which often presents at advanced stages, identifying specific molecular vulnerabilities is crucial for improving therapeutic outcomes [19].

This study adds value to the existing literature in several ways. First, it is among the largest retrospective cohorts from Eastern Europe and the Caucasus, reflecting a multi-center collaboration that enhances generalizability. Second, the integration of detailed IHC analysis with survival outcomes provides novel evidence linking immune microenvironment composition and p53 status to clinical prognosis. Third, the simultaneous demonstration of progressive receptor loss, proliferative escalation, and immune dysregulation across the ESN-LG-ESS-HG-ESS spectrum offers a holistic perspective on tumor progression. These features collectively establish our study as a substantive contribution to both diagnostic pathology and translational oncology. Future research should focus on validating these immunohistochemical panels in prospective trials and exploring targeted therapies that leverage these biological insights, particularly for the high-grade variants.

The practical implications of these findings are several. Hormonal therapy remains an effective adjuvant strategy for LG-ESS, supported by the preserved ER/PR expression in most cases. However, the poor outcomes associated with HG-ESS and their immune-evasive microenvironment suggest that conventional endocrine therapy is inadequate. For these patients, immunotherapy or targeted agents (such as CDK4/6 inhibitors or anti-angiogenic compounds) merit exploration. Additionally, the identification of p53 aberrations as a poor prognostic factor supports the inclusion of molecular profiling in routine diagnostics to guide management decisions. For instance, the aggressive nature of HG-ESS often necessitates chemotherapy, while LG-ESS typically responds well to hormonal interventions such as aromatase inhibitors and progestins [1].

This study is not without limitations. The retrospective design introduces potential selection bias, and while the median follow-up exceeded 4 years, longer observation is needed to fully capture the natural history of LG-ESS, which is prone to late recurrence. Molecular testing was not universally available across cases, limiting the integration of genetic correlates with immunophenotypic findings. Nonetheless, the robustness of our immunohistochemical and clinical data compensates for these constraints and provides a strong foundation for future prospective studies. Future research should also investigate the utility of advanced imaging techniques, such as positron emission tomography–computed tomography (PET-CT) [29], for early detection of recurrence, particularly given the propensity for aggressive behavior in higher-grade variants.

Our findings highlight several avenues for future research. Prospective multi-center studies incorporating next-generation sequencing and transcriptomic profiling could elucidate the biological pathways underpinning receptor loss, immune evasion, and p53 dysregulation. Functional investigations into the crosstalk between tumor cells and M2 macrophages may reveal therapeutic vulnerabilities. Moreover, clinical trials of immunotherapy in HG-ESS, particularly in patients with high CD163^+^ macrophage burden, could establish novel treatment paradigms for this aggressive disease. Furthermore, exploring novel drug combinations targeting both cellular proliferation and immune evasion mechanisms could yield more effective therapeutic strategies against HG-ESS.

### Conclusions

This multi-center retrospective study underscores the biological and clinical heterogeneity of ESTs across the spectrum from ESN to LG-ESS and HG-ESS. Progressive loss of ER/PR expression, increased Ki67, aberrant p53, and enrichment of CD163-positive macrophages were all associated with higher tumor grade and shorter DFS. In multivariable analysis, histological grade, LVSI, p53 status and hormone receptor expression remained independent predictors of recurrence. These findings support the incorporation of proliferative and immune biomarkers into future risk-stratification algorithms and highlight potential targets for individualized therapeutic strategies, particularly in high-grade disease.

## Data Availability

The datasets generated and analyzed during the current study are not publicly available due to patient confidentiality but are available from the corresponding author on reasonable request and with approval from the Institutional Ethics Committee.
